# Influence of the arterial elastic component on the response to balloon angioplasty in femoral arteries of a healthy porcine model

**DOI:** 10.1002/ame2.70024

**Published:** 2025-05-09

**Authors:** María Gracia de Garnica García, Marina Gil Bernabé, Claudia Pérez‐Martínez, Laura Mola Solà, Luis Duocastella Codina, María Molina Crisol, Alex Gómez Castel, Armando Pérez de Prado

**Affiliations:** ^1^ Department of Animal Health, Section of Pathology, Veterinary School University of León León Spain; ^2^ Micros Veterinaria S.L. León Spain; ^3^ iVascular Barcelona Spain; ^4^ Complejo Asistencial Universitario de León León Spain

**Keywords:** angioplasty, elastin, femoral artery, histology, paclitaxel, porcine model

## Abstract

**Background:**

The efficacy of balloon angioplasty for treating peripheral artery disease is influenced by various factors, some of them not yet totally understood. This study aimed to evaluate the role of elastin content in vascular responses 28 days post‐angioplasty using uncoated and paclitaxel‐coated balloons with the same platform in femoral arteries of a healthy porcine model.

**Methods:**

Eight animals underwent balloon angioplasty on the external and internal branches of femoral arteries. Histopathologic evaluation was conducted at follow‐up to assess the elastin content, vascular damage, morphological features, and neointimal formation.

**Results:**

The elastin content was significantly higher in the external than in the internal femoral artery (*p* = 0.0014). After balloon angioplasty, it was inversely correlated with vascular injury score (*ρ* = −0.4510, *p* = 0.0096), neointimal inflammation (*ρ* = −0.3352, *p* = 0.0607), transmural (*ρ* = −0.4474, *p* = 0.0103) and circumferential (*ρ* = −0.4591, *p* = 0.0082) smooth muscle cell loss, presence of proteoglycans (*ρ* = −0.5172, *p* = 0.0024), fibrin deposition (*ρ* = −0.3496, *p* = 0.0499), and adventitial fibrosis (*ρ* = −0.6229, *p* = 0.0002). Neointimal formation inhibition with paclitaxel was evident only in arteries with disruption of the internal elastic lamina, with a significant smaller neointimal area in arteries treated with paclitaxel‐coated balloons compared to uncoated balloons (median [Q1–Q3]: 10.25 [7.49–15.64] vs. 24.44 [18.96–30.52], *p* = 0.0434).

**Conclusions:**

Elastin content varies between branches of the femoral artery and significantly influences the integrity of the internal elastic lamina, the vessel's adaptive response, and paclitaxel efficacy after balloon angioplasty.

## Graphical abstract

This study evaluates the role of elastin in vascular response 28 days after balloon angioplasty using uncoated and paclitaxel‐coated balloons in a healthy porcine model. The results show higher elastin content in external femoral arteries, which is inversely correlated with vascular injury, inflammation, smooth muscle cell loss, proteoglycan and fibrin deposition, and adventitial fibrosis. Additionally, paclitaxel treatment inhibited neointimal formation, but this effect was only observed in arteries with disruption of the internal elastic lamina.

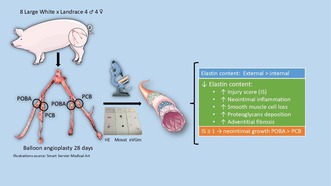

## 1 INTRODUCTION

Peripheral artery disease (PAD) is a prevalent manifestation of atherosclerosis worldwide, affecting up to 10% of adults over 20 years of age.^1^ In patients with advanced symptoms who do not respond to medical therapy, open surgical or endovascular revascularization (EVR) procedures are necessary.^2^ The use of drug‐coated balloons (DCBs) has become the standard EVR technique in treating PAD. This preference arises from the limited use of stents owing to the heightened risk of strut fracture, a consequence of the biomechanical properties of the affected vessels and blood flow.^3^ Additionally, DCBs demonstrate superior efficacy compared to plain old balloon angioplasty (POBA), particularly by significantly reducing the need for target lesion revascularization.^4^

Patient‐related clinical factors, including hypertension, glycemia, renal function, and functional status, are commonly regarded as risk factors that may influence the success of EVR.^5^ However, limited information is available regarding the impact of histologic arterial characteristics, which are key determinants of arterial function, on EVR.^6^ Preclinical animal models are crucial for the development and understanding of treatments for PAD. Among these, the porcine model is the most widely used due to its anatomical and physiological resemblance to human vessels, making it the preferred choice and the most accepted by regulatory authorities.^7^ To advance in the development of DCBs that enable effective, safe, and durable revascularization, it is essential to understand the factors underlying why EVR cannot be directly translated to human applications, despite demonstrating efficacy in specific preclinical models.

In this regard, it is important to evaluate the influence of changes in the extracellular matrix (ECM) components of an artery during balloon angioplasty on the vascular response. Some studies have examined the role of collagen fibers in preventing vessel rupture due to the high pressures reached during angioplasty^8^ and of muscle fibers in understanding the mechanism of dilation.^9^ However, studies focusing on the function of elastic fibers are scarce and not related to EVR procedures.^10^

The aim of this study is to understand the influence of the elastin content on the vascular wall of femoral arteries in a healthy porcine model and its potential effect on the vascular response 28 days after treatment with paclitaxel‐coated balloons (PCBs) or POBAs.

## 2 METHODS

This study was approved by the Animal Research Committee at the Centre de Medicina Comparativa i Bioimatge (Badalona, Spain) under reference number 11379. It was conducted in compliance with Directive 2010/63/EU^11^ and Spanish Royal Decree 53/2013,^12^ which govern the protection of experimental animals.

### 2.1 Study devices

Two coaxial, semi‐compliant, over‐the‐wire balloon catheters designed for the treatment of femoral and popliteal arterial lesions were used in this study. The devices included POBAs and PCBs (containing paclitaxel 3.5 μg/mm^2^) manufactured by iVascular (Barcelona, Spain). Both devices share the same balloon platform, mechanical properties, and inflation pressure. The nominal diameter of all the devices was 6 mm.

### 2.2 Study groups

Eight healthy large white × Landrace pigs (four females and four males), aged 3–4 months and weighing between 30 and 35 kg, were used in this study. These pigs were not subjected to any modifications or specific selection criteria. After a 7‐day acclimatization period, bilateral peripheral balloon angioplasty was performed on the internal and external branches of the femoral arteries. Samples were divided into two groups (Table 1) based on the device applied to each treated femoral branch, using a random allocation method. Sample size was calculated based on previous publications.^13^

**TABLE 1:** Sample allocation and location per study group.

Animal	Sex	Right external femoral artery	Right internal femoral artery	Left external femoral artery	Left internal femoral artery
1	F	PCB	PCB	POBA	POBA
2	F	POBA	POBA	PCB	PCB
3	F	PCB	PCB	POBA	POBA
4	F	POBA	POBA	PCB	PCB
5	M	PCB	PCB	POBA	POBA
6	M	POBA	POBA	PCB	PCB
7	M	PCB	PCB	POBA	POBA
8	M	POBA	POBA	PCB	PCB

*Note*: Samples randomized by random allocation table.

Abbreviations: F, female; M, male; PCB, paclitaxel‐coated balloon; POBA, uncoated balloon or plain old balloon angioplasty.

### 2.3 Procedures

After the induction of general anesthesia, initial bilateral angiography using an IVR Alphenix INFX.8000V/Y3 system (Canon, Tokyo, Japan) was conducted to evaluate the specific vascular territories.

A midline neck incision and dissection of muscular tissue preceded the insertion of an 8F arterial sheath (Terumo, Tokyo, Japan) into the carotid artery. Subsequently, a 6F guiding sheath (Cook, Indiana, USA) was advanced under fluoroscopic guidance to the distal aortic bifurcation. An arterial segment was selected in each femoral branch (internal and external, left and right) on the basis of an adequate reference diameter to achieve a balloon‐to‐artery ratio of 1.1:1 to 1.2:1. The devices were positioned a minimum of 5 mm distal to the proximal bifurcation and were inflated for 1 min within 2 min from insertion. Inflation pressure was adjusted to match the target arterial diameter according to the manufacturer's compliance chart, ensuring consistency across both POBA and PCB treatments. After the four arteries were treated, the entire system was withdrawn, the carotid artery was ligated proximally and distally to the puncture site, and the surgical wound was sutured in layers. An intramuscular injection of atipamezole (0.1 mg/kg, Zoetis, New Jersey, USA) reversed the anesthesia.

Animals were administered daily doses of 75 mg of clopidogrel (Neuraxpharm, Langenfeld, Germany) and 125 mg of acetylsalicylic acid (Opella Healthcare, Madrid, Spain) throughout the 28‐day follow‐up period. Cefquinome (MSD, New Jersey, USA) antimicrobial therapy was applied for 6 days post‐procedure.

### 2.4 Histologic evaluation

After the 28‐day follow‐up period, the animals underwent angiography under anesthesia to assess the treated femoral artery branches. Euthanasia was performed by intravenous administration of a pentobarbital overdose (50–200 mg/kg, Euthoxin 500 mg/mL; Labiana Life Sciences, Barcelona, Spain).

All femoral segments were macroscopically evaluated, flushed with a saline solution, and perfusion‐fixed with 10% buffered formalin. Treated arterial segments were collected along with adjacent untreated segments measuring 1 cm in length, both proximally and distally.

Two regions (treated and untreated) of each sample were trimmed and embedded in paraffin. Three sections, each 2.5 μm thick, were obtained and stained with hematoxylin–eosin (HE), Movat pentachrome, and modified van Gieson (eVGm) stains. This modification facilitates the differentiation of elastic fibers by not contrasting the background with any dye.

eVGm slides were scanned using an Olympus BX51 whole slide scanner (Tokyo, Japan) to analyze the elastin content. Measurements were conducted using QuPath software version 0.5.1^14^ and are expressed as percentages of the area of media, adventitia, and total wall area.

The vascular damage resulting from the procedure was assessed in Movat‐stained slides using the injury scoring (IS) system developed by Schwartz et al.^15^ (Table 2).

**TABLE 2:** Vascular damage injury score^15^ and morphological parameters^16^ scoring system.

Scored parameter	0	1	2	3	4
Vascular damage					
Injury score	Intact internal elastic lamina	Lacerated internal elastic lamina Tunica media compressed	Lacerated internal elastic lamina External elastic lamina compressed	Lacerated external elastic lamina	NA
Inflammation					
Intima/media	None	<20 inflammatory cells/HPF in <25% of area	21–100 inflammatory cells/HPF in 25%–50% of area	101–150 inflammatory cells/HPF >51%–75% of area	>150 inflammatory cells/HPF >75% of area
Inflammation adventitia	None	<20 inflammatory cells/HPF in <25% of area	21–100 inflammatory cells/HPF in 25%–50% of area	101–150 inflammatory cells/HPF >51%–75% of area	>150 inflammatory cells/HPF >75% of area
Medial smooth muscle loss					
Medial SMC loss (transmural)	None	Smooth muscle loss <25% of medial thickness	25%–50% of medial thickness	51%–75% of medial thickness	>75% of medal thickness
Medial SMC loss (circumferential)	None	<25% of the area	25%–50%	51%–75%	>75%
Medial smooth muscle cell replacement tissue					
Medial SMC replacement fibrin	None	<25% of the area	25%–50% of area	51%–75% of area	>75% of area
Medial SMC replacement proteoglycans	None	<25% of the area stains gray‐blue with H&E	25%–50% of area stains gray‐blue with H&E	51%–75% of area stains gray‐blue with H&E	>75% of area stains gray‐blue with H&E
Fibrosis					
Fibrosis in adventitia	None	Minimal, focal	Mild, multifocal	Moderate, regionally diffuse	Severe, marked diffuse, or total luminal occlusion

Abbreviations: H&E, hematoxylin–eosin; HPF, high power field; NA, not applicable; SMC, smooth muscle cell.

Additionally, morphological evaluation, including the transmural loss of smooth muscle cells (SMC), the circumferential SMC loss, the replacement of SMC with fibrin and proteoglycans (PG) in the media, and the degree of adventitial fibrosis, was carried out according to the semiquantitative scoring system described by Yazdani et al.^16^ Inflammation was assessed in HE‐stained slides.

Histomorphometric analysis was performed in Movat‐stained slides using ImageJ software (version 1.54a, National Institutes of Health, Bethesda, MD, USA), measuring the luminal area (mm^2^), internal elastic lamina (IEL) area (mm^2^), external elastic lamina (EEL) area (mm^2^), and vessel boundary area (mm^2^), defined at the margin of the dense adventitial connective tissue. These measurements were used to calculate the neointimal area (N = IEL area – luminal area, mm^2^), neointimal growth ([1 – (luminal area/IEL area)] × 100, %), medial area (M = EEL area – IEL area, mm^2^), adventitial area (A = vessel boundary area – EEL area, mm^2^), and total wall area (medial area + adventitial area, mm^2^).

The macroscopic and histological evaluation of the samples was conducted under blinded conditions.

### 2.5 Statistical analysis

Statistical analyses were performed to evaluate differences and relationships between variables. Continuous variables were tested for normality using the Shapiro–Wilk test, and group differences were assessed using either Student's *t*‐test or the Wilcoxon rank‐sum test, depending on the data distribution. Correlation analyses were performed using Spearman's correlation coefficient (*ρ*) for associations between continuous and ordinal variables, Kendall's correlation coefficient (*τ*) for associations between two ordinal variables, and biserial correlation (*r*_bis_) for associations between a continuous and a dichotomous variable. Statistical significance was set at *p* < 0.05, with values between 0.05 and 0.1 considered as trends. Analyses were performed using R version 4.03 (GNU GPL, Austria).

## 3 RESULTS

All animals survived the 28‐day follow‐up period. No animals or arteries were excluded from the study.

**FIGURE 1:**
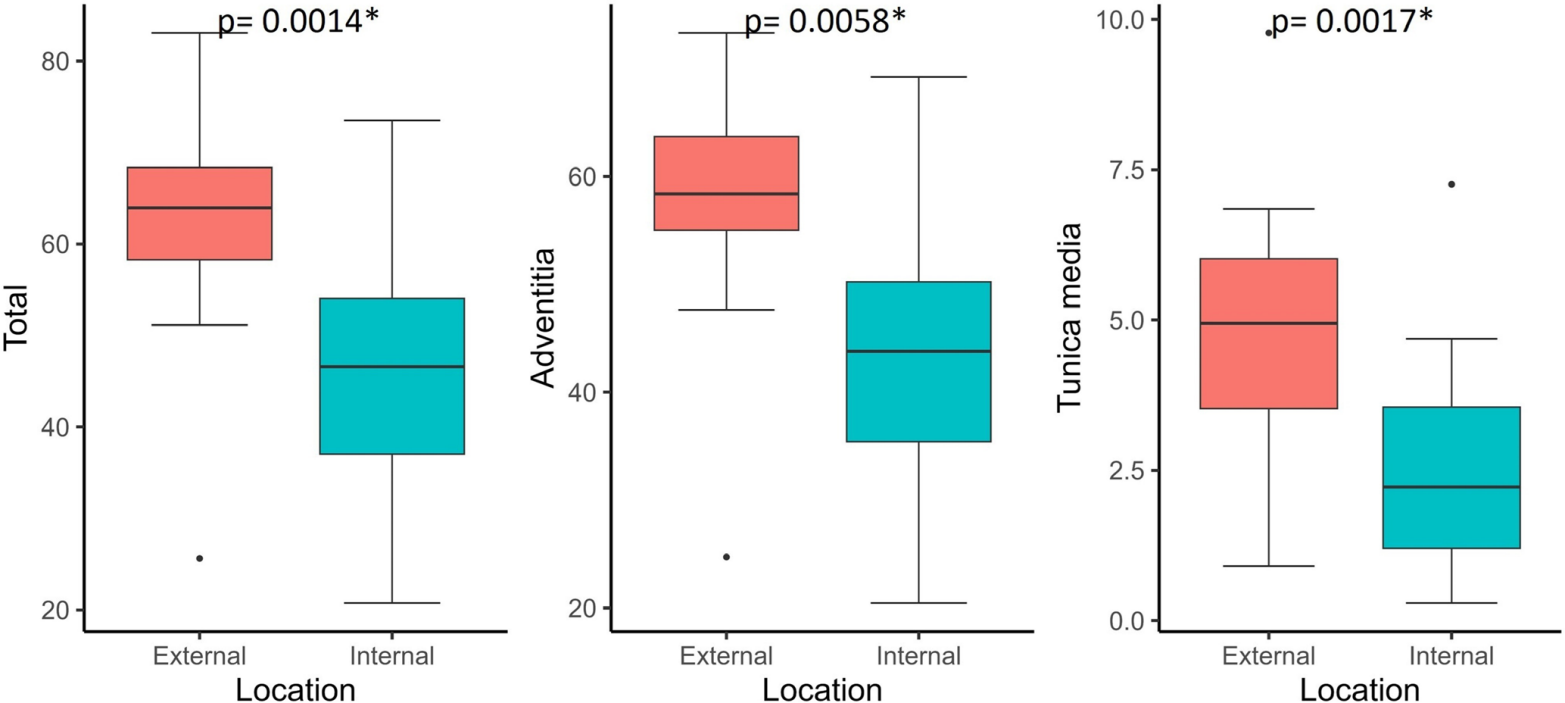
Boxplot comparing the total, adventitial, and tunica media elastin content between external and internal femoral arteries in the untreated segment. Statistically significant differences between arteries are indicated with an asterisk (*).

**FIGURE 2:**
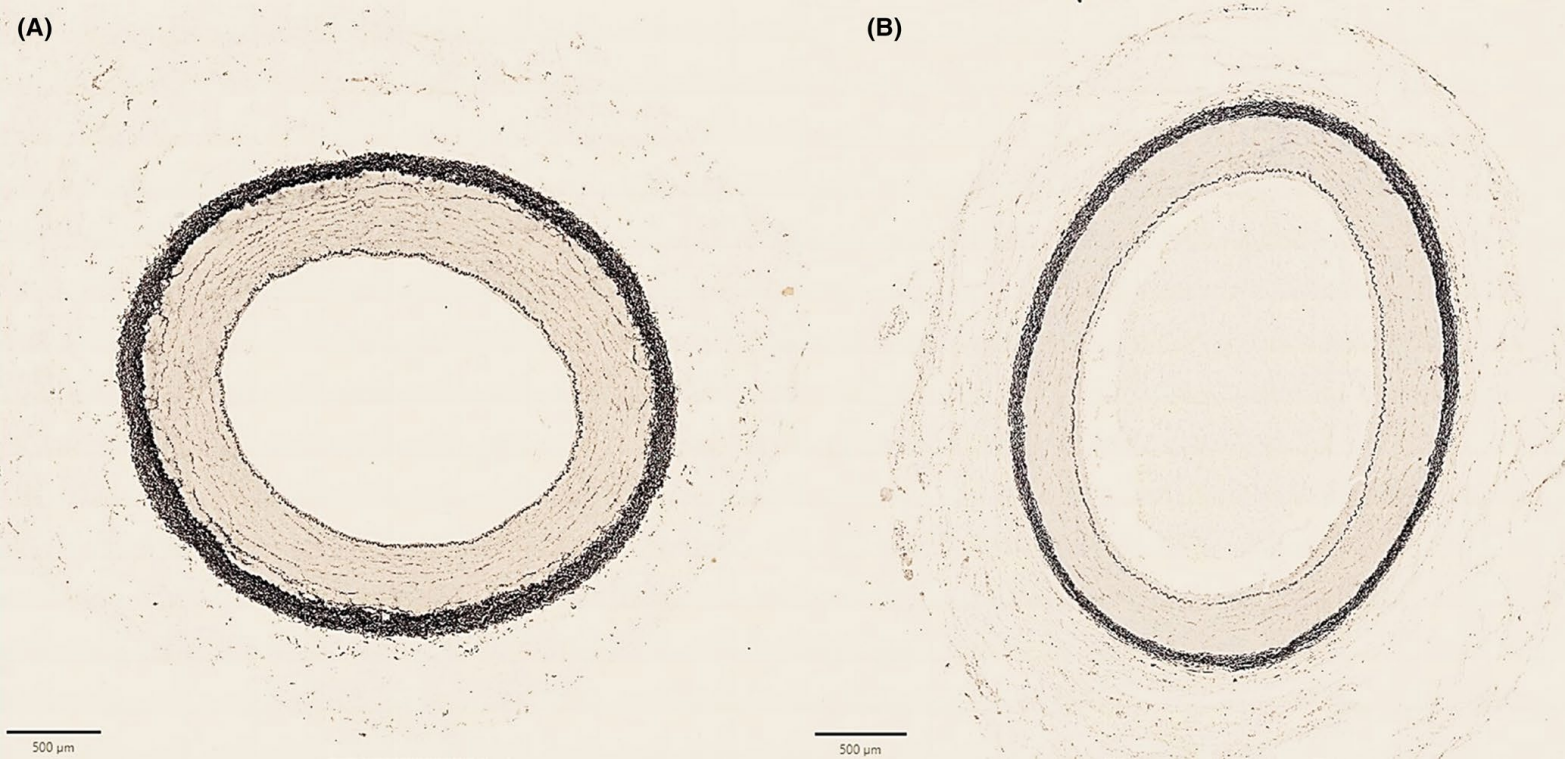
Animal 6. Transverse sections of untreated levels of the (A) external femoral artery and (B) the internal femoral artery stained with van Gieson's elastin‐modified stain. The external femoral artery exhibits a higher density of elastic fibers compared to the internal femoral artery. Scale bar: 500 μm.

The external femoral arteries showed statistically significant higher elastin content (in total, adventitia, and media) than the internal femoral arteries (Figures 1, 2A,B).

### 3.1 Vascular damage and morphological evaluation

**FIGURE 3:**
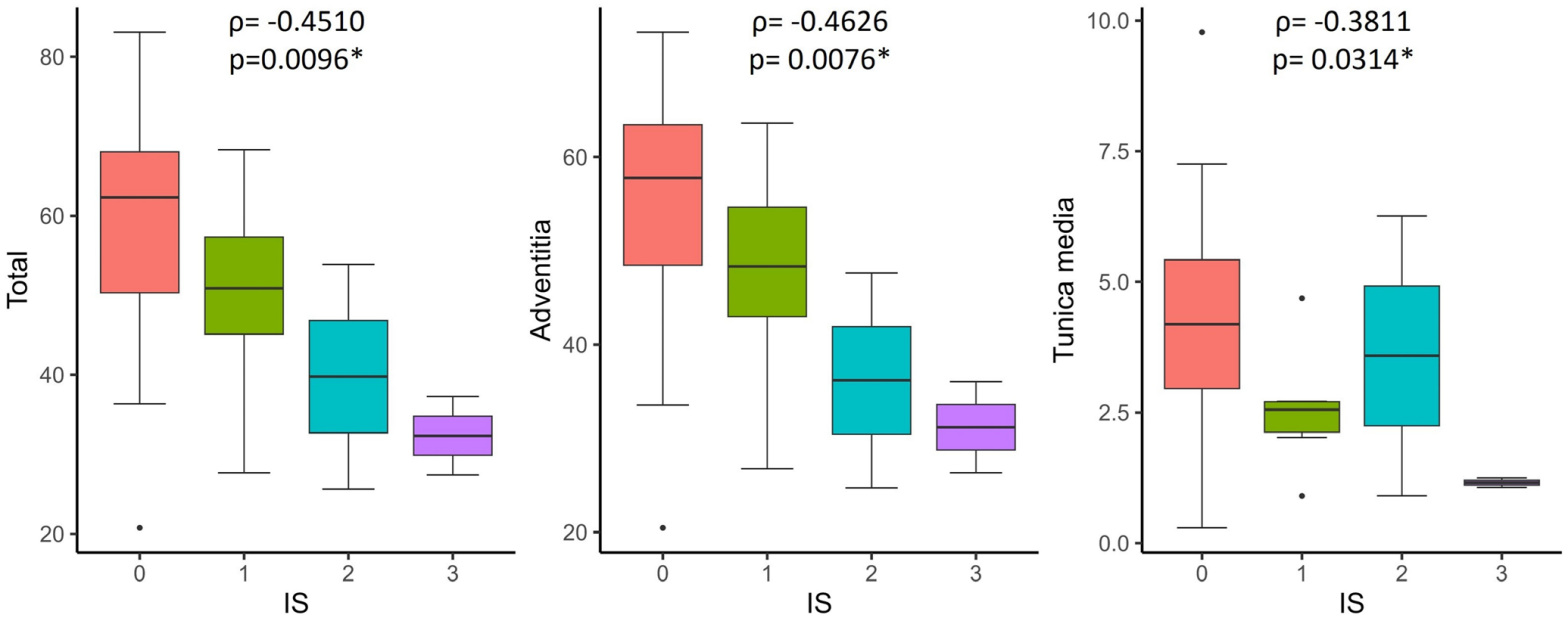
Boxplot showing the total, adventitial, and tunica media elastin content in the untreated segment per grade of injury score (IS). The Spearman correlation coefficient (*ρ*) and its statistical significance (*p*) are indicated for each analysis. Statistically significant associations are marked with an asterisk (*).

Total elastin content, including both adventitial and the medial content, showed an inverse association with the IS (Figure 3).

Neointimal inflammation was observed exclusively in PCB‐treated arteries, with 75% (12/16) showing no inflammation (score 0), 18.75% (3/16) exhibiting mild inflammation (score 1), and 6.25% (1/16) presenting severe inflammation (score 3). In contrast, all POBA‐treated arteries (16/16, 100%) had no inflammation (score 0), showing a statistically significant difference between groups (*p* = 0.0386). Regardless of the treatment, neointimal inflammation was inversely associated with the media elastin content (*ρ* = −0.3430, *p* = 0.0470), whereas the adventitial and total elastin content showed a trend toward this association (*ρ* = −0.3352, *p* = 0.0607 for both). In PCB‐treated arteries, neointimal inflammation showed a trend toward a negative association with total, adventitial, and tunica media elastin content (*ρ* = −0.4292, *p* = 0.0972 for all), and positively associated with IS (*τ* = 0.6064, *p* = 0.0110).

The morphological features evaluated in the tunica media revealed no significant differences between treatments with respect to transmural and circumferential SMC loss or PG deposition. Fibrin deposition was observed only in PCB‐treated samples. The evaluation between treated and untreated arterial levels found differences in transmural SMC loss (*p* = 0.0414) and a trend toward differences in circumferential SMC loss (*p* = 0.953) exclusively in PCB‐treated samples.

**FIGURE 4:**
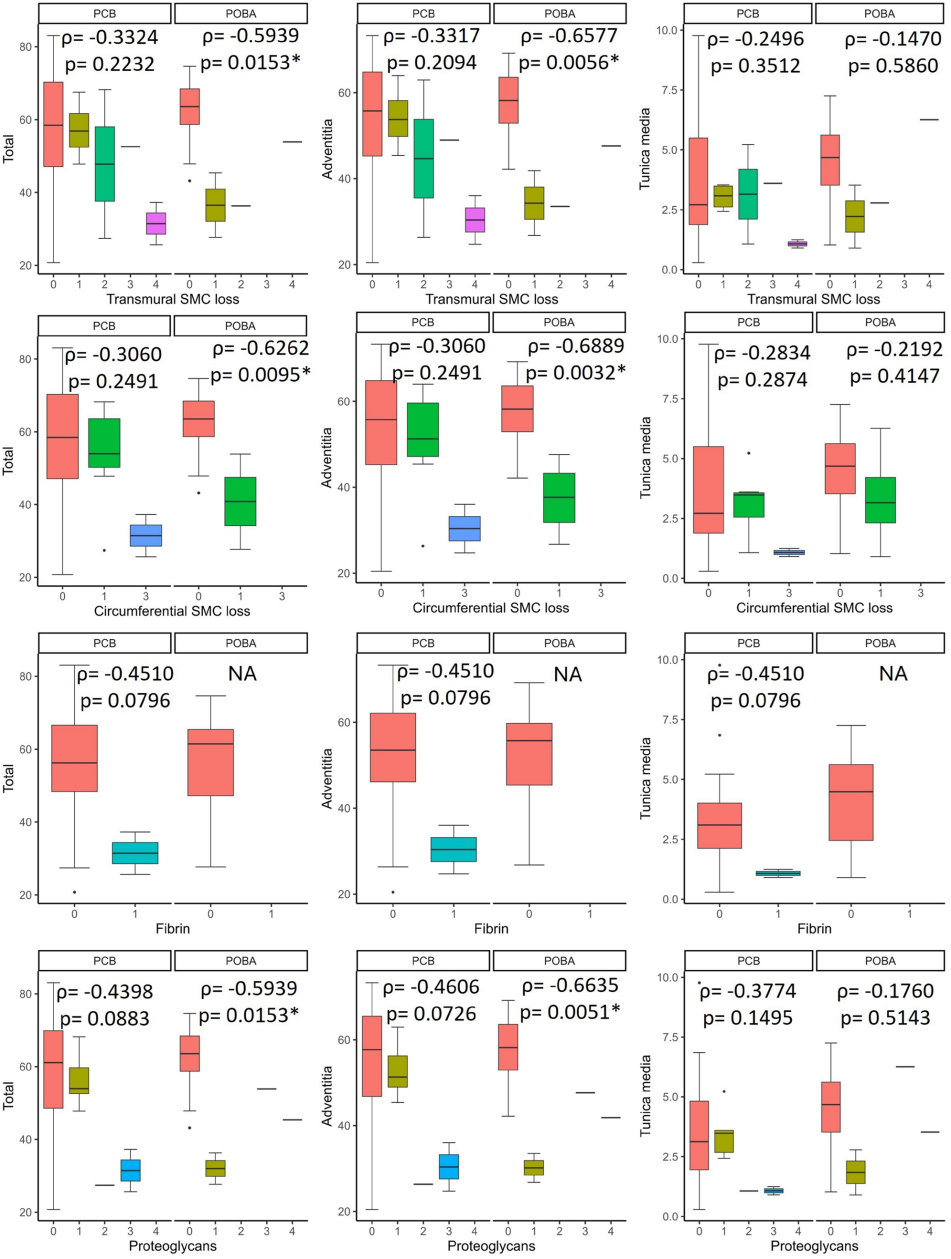
Boxplot showing the total, adventitial, and tunica media elastin content in the untreated segments per grade of transmural and circumferential smooth muscle cell (SMC) loss, and fibrin and proteoglycan deposition according to the semiquantitative scoring system described by Yazdani et al.^16^ Spearman correlation coefficient (*ρ*) and its statistical significance (*p*) are indicated for each analysis. Statistically significant associations are marked with an asterisk (*).

An analysis performed independently of treatment revealed results similar to those observed with POBA. When analyzed by treatment, POBA‐treated arteries showed an inverse association between total and adventitial elastin content and both transmural/circumferential SMC loss and PG deposition. In PCB‐treated samples, total and adventitial elastin content showed a trend toward an inverse correlation with fibrin and PG deposition, and media elastin content displayed a similar trend with fibrin deposition (Figure 4).

In the adventitia, fibrosis was inversely associated with total and adventitial elastin content (*ρ* = −0.6229, *p* = 0.0002 and *ρ* = −0.6257, *p* = 0.0002, respectively), whereas the tunica media elastin content showed a trend toward this association (*ρ* = −0.4842, *p* = 0.0558). Regarding inflammation in the adventitia, no association was found with the elastin content, but it was positively associated with the IS (*τ* = 0.2965, *p* = 0.0851).

### 3.2 Histomorphometric analysis

**FIGURE 5:**
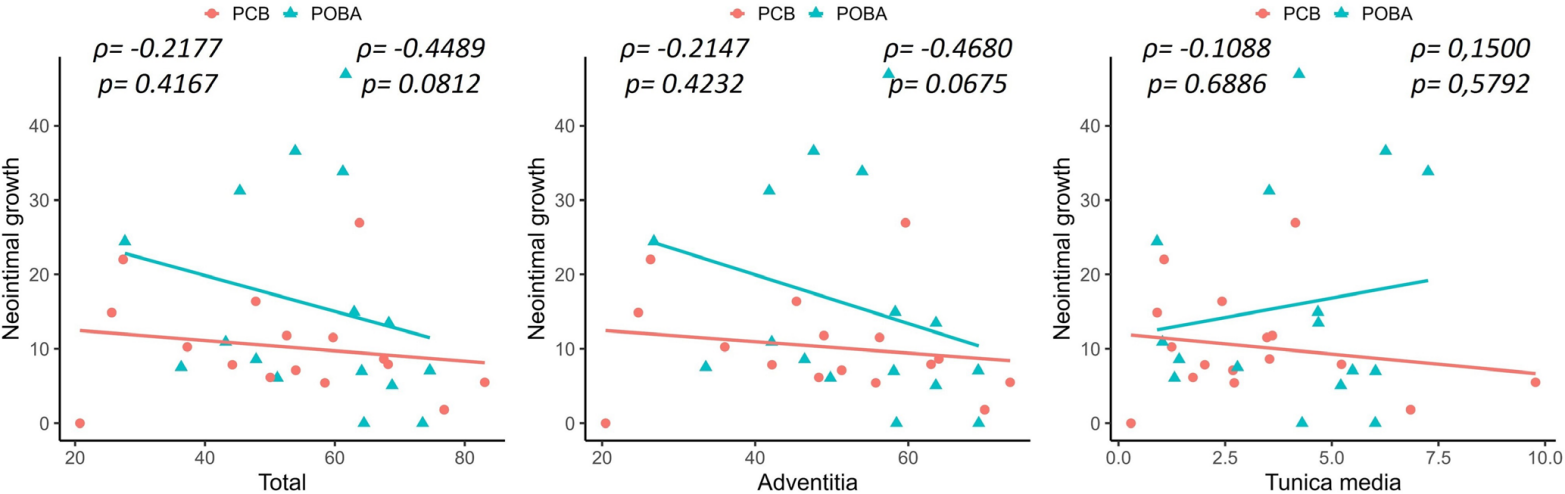
Scattered plot showing the relationship between the total, adventitial, and tunica media elastin content in the untreated segments and the neointimal growth. Spearman's correlation coefficient (*ρ*) and its statistical significance (*p*) are indicated for each analysis. Trend lines illustrate the correlation direction for each group: Paclitaxel‐coated balloon (PCB, red circles) and uncoated balloon (POBA, green triangles).

**FIGURE 6:**
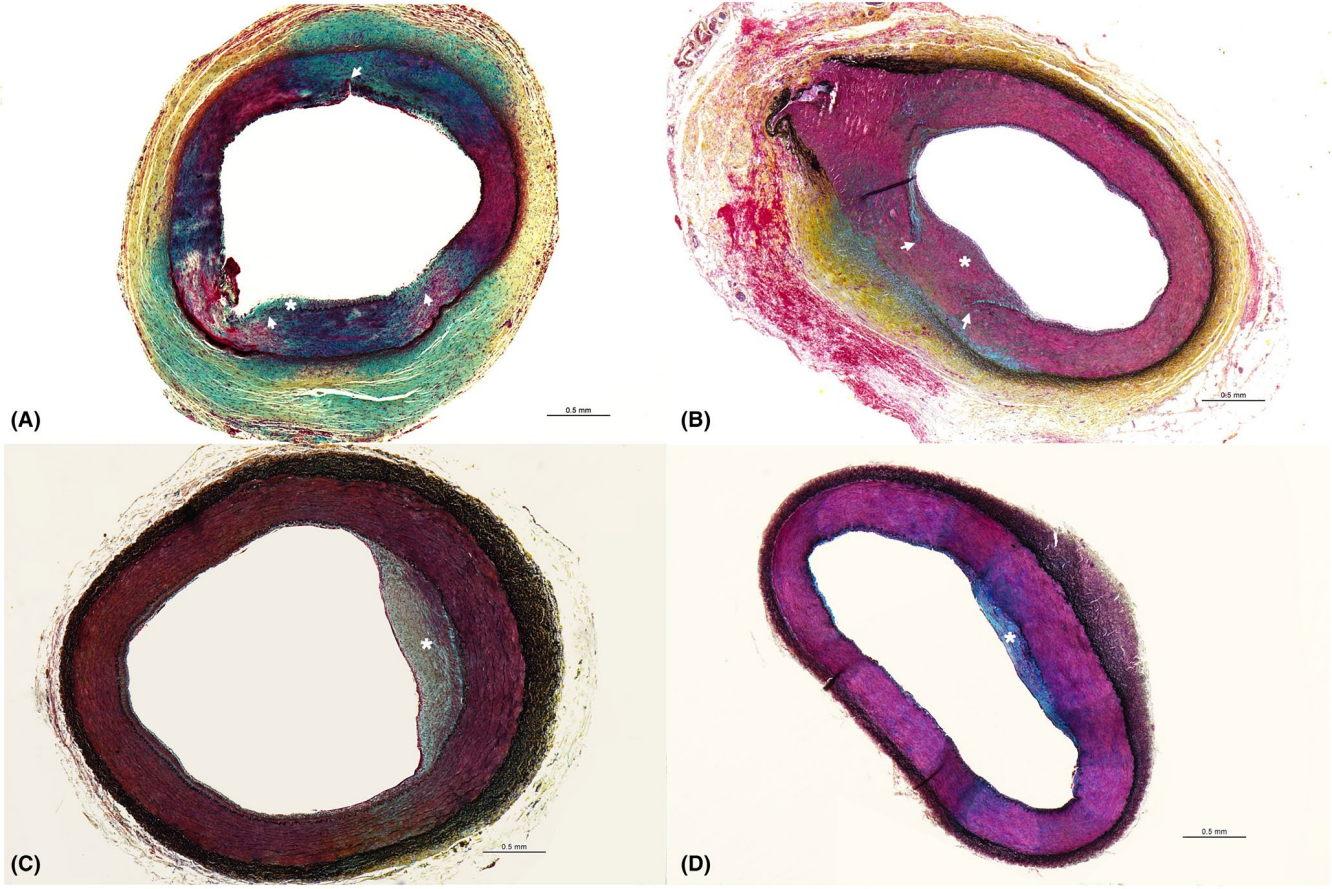
Representative Movat‐stained histological sections demonstrating neointimal formation (*) and vascular injury (→). (A) Animal 4, left internal femoral artery (paclitaxel‐coated balloon [PCB]): Minimal neointimal formation with vascular damage (injury score [IS] ≥ 1), showing proteoglycan deposition in the tunica media and adventitia (blue) and occasional fibrin (bright red). (B) Animal 4, right internal femoral artery (plain old balloon angioplasty [POBA]): Marked neointimal formation in the area with internal elastic lamina (IEL) rupture (IS ≥1). (C) Animal 3, right internal femoral artery (PCB): Neointimal formation with preserved IEL. (D) Animal 8, right internal femoral artery (POBA): Neointimal formation with preserved IEL.

Neointimal growth showed a trend toward an inverse association with total elastin content (*ρ* = −0.3135, *p* = 0.0806), as well as with adventitial elastin content (*ρ* = −0.3326, *p* = 0.0629). By treatment group, this trend toward association with the total elastin fiber content and the adventitial fibers was observed only in the POBA‐treated samples, but not in the PCB‐treated arteries (Figure 5). The biserial correlation revealed a significant relationship between neointimal growth and treatment in samples with IS ≥1 (*r*_bis_ = −0.6464, *p* = 0.0434), resulting in significantly lower neointimal growth in PCB‐treated (median [Q1–Q3]: 10.25 [7.49–15.64]) than in POBA‐treated arteries (median [Q1–Q3]: 24.44 [18.96–30.52]) with IEL rupture (*p* = 0.0434) (Figure 6A,B). Conversely, no such relationship nor differences between PCB‐ and POBA‐treated arteries were observed in samples with IS = 0 (Figure 6C,D).

## 4 DISCUSSION

The main findings of this study indicate that in the healthy porcine model, the elastin content was (a) higher in the external femoral arteries compared to the internal femoral arteries; (b) inversely associated with markers of vascular injury, such as IS, SMC transmural and circumferential loss, PG and fibrin deposition, and adventitial fibrosis; and (c) showed a trend toward association with neointimal inflammation and neointimal growth. Finally, the effect of drug treatment on neointimal growth control was observed only in samples exhibiting rupture of the IEL (IS ≥1).

In our study, internal femoral arteries exhibited significantly lower elastin content than external femoral arteries in the healthy porcine model. Changes in the quantity or organization of elastic fibers result in alterations in the arterial structure and mechanical properties.^17^ Accounting for this feature may aid in interpreting discrepancies between studies applying the same treatment and could also explain why certain treatments proven effective in animal models do not translate as successfully to human applications.^18^ Therefore, we propose that this specific characteristic of the femoral arteries should be considered in the planning of preclinical studies using this model.

Furthermore, these differences suggest the possibility that the anatomical location of the lesion may influence the vessel response to balloon‐induced distension, potentially affecting treatment efficacy.^19^ In this context, optical coherence tomography (OCT) could serve as a valuable tool for assessing the elastic component of vascular structure,^20^ providing an additional parameter to consider when planning these procedures for each patient.

In our study, we confirmed that lower elastin content in the arterial wall was associated with higher IS, consistent with previous findings.^21^ Higher degree of IS is associated with a more pronounced inflammatory response,^22^ which promotes the vascular repair process and results in more severe neointimal growth.^23^ Our results demonstrate a positive correlation between the IS and neointimal inflammation. These findings indicate that the anatomical location of the lesion, along with the subsequent variation in elastin content, may influence the vessel's response to balloon‐induced distention, potentially affecting treatment efficacy.

Because both PCB and POBA were based on the same platform and inflated at a consistent pressure according to the manufacturer's compliance chart, the observed differences between treatments can be attributed to the effect of paclitaxel.

An inverse association was observed between elastin content and transmural and circumferential SMC loss, as well as PG deposition, in the POBA‐treated arteries, suggesting that it results from the mechanical stress induced by angioplasty. PGs, which are abundant in the ECM of the vessel wall, play a critical role in the vascular remodeling after angioplasty. Their deposition is linked to lipoprotein retention, SMC migration, and ECM remodeling, which contribute to neointimal formation and wall thickening.^24^ Fibrin deposition was observed exclusively in PCB‐treated arteries, where a trend toward an inverse association with elastin content was noted. The presence of fibrin is associated with effective paclitaxel delivery, as the inhibition of neointimal growth is accompanied by fibrin deposition.^25^ This observation highlights the potential influence of elastin content on the effectiveness of paclitaxel delivery within the vessel wall.

The inverse relation between elastin content and adventitial fibrosis emphasizes the essential role of elastin in mitigating constrictive vascular remodeling. This process, driven by myofibroblast proliferation within the adventitia, is a significant contributor to the lumen loss following balloon angioplasty.^26^ Moreover, the association found between the IS and adventitial inflammation, which promotes fibroblast activation in the adventitia, further underlines the protective role of elastin in preventing the constrictive vascular remodeling process.^27^

We observed that the neointima of the femoral arteries subjected to the procedure was more pronounced in vessels with lower elastin content. This effect is likely associated with the vascular damage inflicted during the intervention, which has been shown to have a direct relationship with neointimal formation.^28^

Moreover, an association was observed between the control of neointimal growth and treatment only in arteries with rupture of IEL (IS ≥1). This can be attributed to the dual role of elastin in the vascular response. Elastin not only contributes to vascular elasticity but also serves as a substrate for binding lipophilic substances such as paclitaxel, owing to its hydrophobic properties.^29^ Even minimal amounts of elastin can act as a substantial barrier to drug transport.^29^ This evidence indicates that, as previously demonstrated in coronary arteries in the same animal model,^30^ the rupture of the IEL is a key determinant of the efficacy of PCB angioplasty. Additionally, considering the association of IS with the elastin content, the amount of elastin may also play a crucial role in modulating PCB angioplasty outcomes.

The findings of this study suggest that the arterial elastic component should be considered in the evaluation of the vascular response of the atherosclerotic arteries to balloon angioplasty. Atherosclerosis is a progressive vascular inflammatory disease characterized by endothelial dysfunction, lipid accumulation, inflammation, and ECM remodeling, all of which compromise the arterial elastic component. Particularly, calcium and lipid deposition and their binding to elastin increase its susceptibility to proteolytic degradation. Additionally, elevated elastase activity accelerates elastin depletion.^31^ Given that proteolytic damage to elastin is considered irreversible, the resulting loss of elastic content in atherosclerotic arteries might contribute to exacerbate vascular damage, inflammation, smooth muscle cell loss, proteoglycan and fibrin deposition, adventitial fibrosis, and stenosis on the response to balloon angioplasty compared to healthy arteries.

This study has a significant limitation: the animal model consisted of healthy, young subjects with no preexisting vascular disease nor induced injuries. These factors do not fully replicate the elastin alterations and vascular conditions commonly observed in human patients with PAD.

## 5 CONCLUSIONS

In conclusion, the femoral arteries exhibit elastin content dependent on their anatomical location in the healthy porcine model. In addition, a lower elastin content is associated with increased IS and various parameters linked to neointimal growth and vascular remodeling following balloon angioplasty in femoral arteries. Based on these findings, we emphasize the importance of considering the anatomical location and elastin content of arteries when designing preclinical studies to evaluate the efficacy of angioplasty devices, as this may help mitigate the differences in efficacy observed when translating results to human patients.

## AUTHOR CONTRIBUTIONS

**María Gracia de Garnica García:** Formal analysis; investigation; visualization; writing – original draft. **Marina Gil Bernabé:** Investigation. **Claudia Pérez‐Martínez:** Conceptualization; investigation; methodology; supervision; validation; visualization; writing – original draft. **Laura Mola Solà:** Conceptualization; investigation; methodology; resources; validation; writing – review and editing. **Luis Duocastella Codina:** Funding acquisition; project administration; supervision. **María Molina Crisol:** Conceptualization; methodology; resources; validation; writing – review and editing. **Alex Gómez Castel:** Conceptualization; methodology; resources; validation; writing – review and editing. **Armando Pérez de Prado:** Conceptualization; data curation; formal analysis; investigation; methodology; supervision; writing – review and editing.

## FUNDING INFORMATION

This study was funded by iVascular, S.L.U., Camí de Can Ubach, 11 – Nave 3, 08620 Sant Vicenç dels Horts, Barcelona, Spain, which also contributed to the study design and the decision to submit the manuscript for publication.

## CONFLICT OF INTEREST STATEMENT

The authors declare that they have no competing interests relevant to the content of this article. Additionally, each author has individually informed the publisher of any professional relationships that could potentially constitute a conflict of interest.

## ETHICS STATEMENT

This study was approved by the Animal Research Committee at the Centre de Medicina Comparativa i Bioimatge (Badalona, Spain) under reference number 11379. EU standards (Directive 2010/63/EU) and national standards (Spanish Royal Decree 53/2013) for the protection of animals used for scientific purposes were met in this study.
